# Starchy Carbohydrates in a Healthy Diet: The Role of the Humble Potato

**DOI:** 10.3390/nu10111764

**Published:** 2018-11-14

**Authors:** Tracey M. Robertson, Abdulrahman Z. Alzaabi, M. Denise Robertson, Barbara A. Fielding

**Affiliations:** Department of Nutritional Sciences, University of Surrey, Guildford, GU2 7WG, UK; t.m.robertson@surrey.ac.uk (T.M.R.); a.alzaabi@surrey.ac.uk (A.Z.A.); m.robertson@surrey.ac.uk (M.D.R.)

**Keywords:** potato, obesity, satiety, T2DM, CVD, nutrition, resistant starch, fibre

## Abstract

Potatoes have been an affordable, staple part of the diet for many hundreds of years. Recently however, there has been a decline in consumption, perhaps influenced by erroneous reports of being an unhealthy food. This review provides an overview of the nutritional value of potatoes and examines the evidence for associations between potato consumption and non-communicable diseases. Potatoes are an important source of micronutrients, such as vitamin C, vitamin B6, potassium, folate, and iron and contribute a significant amount of fibre to the diet. However, nutrient content is affected by cooking method; boiling causes leaching of water-soluble nutrients, whereas frying can increase the resistant starch content of the cooked potato. Epidemiological studies have reported associations between potato intake and obesity, type 2 diabetes and cardiovascular disease. However, results are contradictory and confounded by lack of detail on cooking methods. Indeed, potatoes have been reported to be more satiating than other starchy carbohydrates, such as pasta and rice, which may aid weight maintenance. Future research should consider cooking methods in the study design in order to reduce confounding factors and further explore the health impact of this food.

## 1. Introduction

According to current UK government guidelines, carbohydrate (CHO) intake should be maintained at a population average of approximately 50% of total energy intake [1] and this is strongly supported by a recent meta-analysis indicating that a carbohydrate intake of 50–55% is optimal [2]. The intake of free sugars within the recommendation should not exceed 5% [1]. This is broadly in line with the WHO Scientific Update on carbohydrates in human nutrition (2007) which recommends a minimum of 50% of total energy intake from CHO, with free sugars restricted to <10% [3]. It is further recommended that carbohydrates consist mainly of starchy foods, such as potatoes, pasta, rice and bread, at about one-third of our total food intake [4].

**Figure 1:**
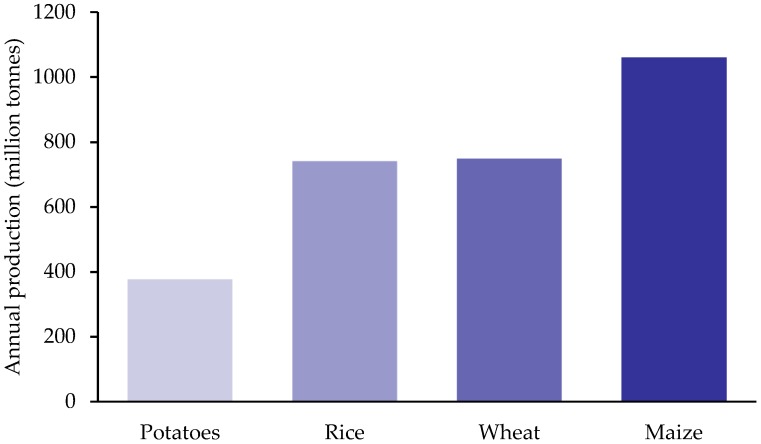
Global production of major starchy carbohydrate crops in 2016 [10].

The potato is historically a starch-rich staple food, originating over 7000 years ago in Peru as reviewed [5]. Potatoes have been an important, affordable food in our diet for hundreds of years and the economic and health consequences of the Irish potato famine between 1845 and 1849 are widely known. As a staple food, the potato still plays an important role in global food security, providing a sustainable food supply and lessening poverty and malnutrition in many parts of the world as highlighted in the Food and Agriculture Organization of the United Nations (FAO) review ‘International Year of the Potato’ [6]. Sustainability of a crop is partly determined by the area of land required, and the water and energy requirements. A ton of potato produced requires only 0.06 ha of land, while rice and wheat require 0.24 and 0.35 ha of land, respectively [7]. Moreover, potato and wheat need less water compared to rice [7] and despite the potato having the highest water content (80%), the energy produced per litre of water is greatest for the potato. In addition, the potato has the lowest carbon footprint of the three [7]. As a crop, potatoes require cool, but frost-free conditions, suiting many geographical areas. However, during storage, potatoes, require chilling and ventilation, which increases the demand for energy [5]. Most cultivated varieties are of the species *Solanum tuberosum* [8], and over the last 60 years, plant biotechnology has complemented conventional potato breeding resulting in specific genotypes or cultivars [9]. The potato is a globally important crop, with an estimated 377 million tonnes harvested in 2016, only falling short of the other starch staples, maize, wheat, and rice (Figure 1).

**Figure 2:**
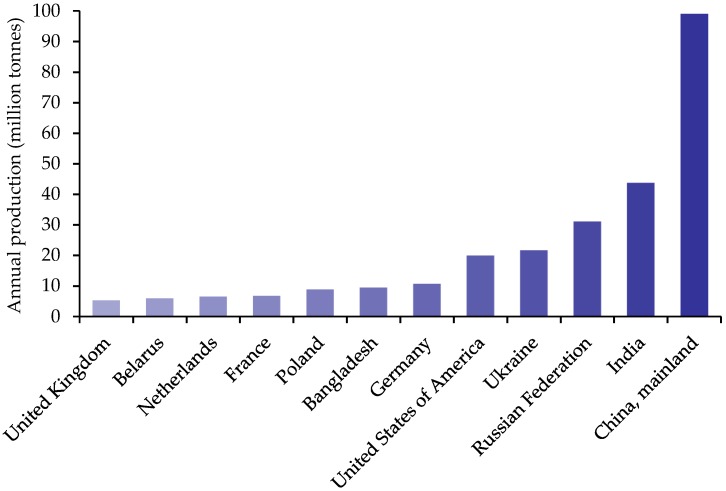
Top twelve producers of potato by country in 2016 [10].

China is estimated to produce the most potatoes in the world with many European countries in the top twelve (Figure 2).

**Figure 3:**
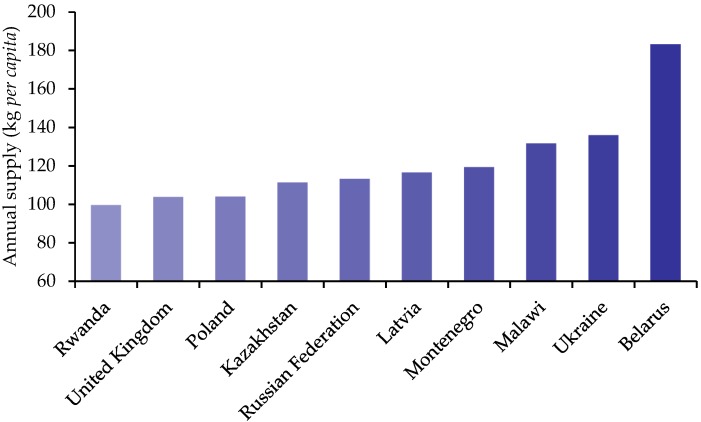
Annual per capita supply of potatoes, available for food, in 2013, as a marker of potential consumption [10]. Figures estimated based on the amounts produced, exported and imported, with deductions made for losses during storage and transport and amounts used for seed, animal feed, and non-food uses.

Many European countries are also in the top ten worldwide potato consumers, when potato supply per capita is used as an estimate of consumption (Figure 3).

Annual per capita data from 2013 also shows that potatoes and potato products are the third most consumed in the diet, behind wheat and rice [10]. Although maize rates higher in terms of world production, it is used in large quantities as a raw material for the manufacture of glucose, fructose, and high glucose corn syrup, as animal feed, and is also increasingly used for industrial applications [11].

Despite the current recommendations for starch in the diet, the nutritional value of the potato could easily be overlooked, partly because it is not counted towards the ‘five-a-day’ fruit and vegetable intake recommendation [4] and because it is often prepared with fats or oils. Indeed, as far back as 1918, the popularity of the potato was attributed to the fact that ‘the lack of flavour makes it possible to confer palatability upon it by the addition of milk, butter, and cream, salt and pepper, or by frying in fats’ [12]. In a UK survey from 2008–2011, potatoes were found to contribute 7% of energy intake [13]. However, potatoes have recently lost favour with an 8.8% reduction in intake from 2013 to 2016/17 in the UK [14]. The reasons for the decline in potato consumption are varied and include changes in food preferences [7]. Potato consumption was recently examined in a Norwegian cohort of women who reported a 15% reduction in consumption from 1998 to 2005 [15]. Increase in income and a perceived association of potato consumption with weight gain and chronic diseases like type 2 diabetes mellitus (T2DM) have been identified as some of the factors responsible for the change, although low-carbohydrate, weight-reducing diets have given conflicting results [16]. Moreover, an increase in prevalence of T2DM has also been identified as a factor leading to a reduction in intake because of dietary advice [15]. However the nutritional benefits of the potato include a relatively high content of micronutrients such as potassium, vitamins such as the B vitamins, and fibre if the skin is eaten [13].

There have been many reviews on potatoes and health in the past; systematic reviews and meta-analyses have covered specific diseases, or focused on specific nutrients, or examined cooking methods [17,18,19,20,21,22]. In this Narrative Review, we have brought these aspects together under the remit of this special edition. In doing so, we have placed our emphasis on the associations between potato intake and non-communicable diseases, such as obesity, cardiovascular disease (CVD), and T2DM. The association between dietary potato intake and health is complex because of confounders such as cooking method, variety, and storage. We will evaluate the effect of such confounders on satiety and metabolic response, and to what extent these have been accounted for in the literature.

## 2. Nutrient Composition

### 2.1. Macronutrients

#### 2.1.1. Carbohydrate

The starch content of a potato can be highly variable. In general terms fresh potatoes contain ~20% dry matter (DM) of which 60–80% is starch, with 70–80% of this starch as amylopectin [23]. This variability is primarily the result of genotype and growing environment.

#### 2.1.2. Fibre

Dietary fibre (DF) is a mixed group of heterogeneous compounds, for the most part, as carbohydrate polymers and oligomers. All definitions identify DF as materials that escape digestion in the small intestine and pass into the large intestine, where they will be fermented by the resident microbiota to a variable extent. DF and their fermentation products, specifically the short-chain fatty acids may contribute many of the beneficial effects of DF consumption for the host. Although there is evidence for the metabolic benefit of DF ingestion from nutritional epidemiology, intervention studies, animal studies and in vitro work, it has mostly been assumed that all DF which share basic physiochemical features; such as solubility, monomeric unit or even botanical source will behave in an identical way, physiologically speaking. However, evidence suggests that different DF may also provide unique properties. The DF composition of potatoes is made up of resistant starch (RS) (major component, see Section 2.1.2.1), with smaller amounts of non-starch polysaccharides such as cellulose (0.45–0.7%) [24], hemi-cellulose (0.32–0.46%), lignin (0.15–0.22%), and pectin (0.32–0.38%) of raw potato mass [25].

The individual fraction responsible for most of the variability in DF content is the RS. Potato tubers are always cooked before consumption so DF values are not typically provided in databases as they are of potentially limited value, however DF values are known to be affected by cooking and serving temperature and typically multiple different values for DF for “potato” will be provided. A major limitation of many epidemiological studies is both the lack of detail on food preparation methods collected as part of the dataset and issues with the laboratory measurement of RS. Despite this obvious limitation, according to the most recent National Diet and Nutrition Survey (NDNS 2015–2016), it is estimated that “potatoes and potato products” contribute ~11% of the AOAC DF intake in adults (19–64 years) in the UK, contributing ~2 g/day [26]. In comparison with other starchy CHO, it contributes less DF than bread (total: ~20%), but more than “pasta, rice, pizza, and other miscellaneous cereals” (8%).

##### 2.1.2.1. Resistant Starch

Resistant starches are the sum of both intact starch and starch degradation products that reach the large intestine for fermentation. The molar yield of butyrate produced by the gut microbiota differentiated RS from other DF fractions [27,28]. RS can be classified into five subtypes; namely, physically entrapped starch (RS1), raw starch granules (RS2), retrograded starch (RS3), chemically modified starch (RS4), and amylose-lipid complex (RS5). The starch in a raw potato tuber is ~75% RS2, with granules resistant to enzyme digestion [29,30]. Data on the exact RS2 content of raw potato tubers is sparse as potatoes would not normally be consumed raw, but estimates are in the region of 47–59% of DM, dependent on variety [29,30]. Thus, an “average” raw potato would contain 10 g RS/100 g wet weight. Potatoes are cooked before consumption and when the starch is heated in excess water gelatinization occurs and the starch becomes highly digestible. Both heat source and water have an impact on this process and following cooking the residual RS2 remaining in the cooked product is relatively low (2–4% DM) but consistently follows the following hierarchy: baked > microwaved > boiled. Cooking time and intensity also affect the amount of RS in the cooked potato, with lower temperature and longer cooking time resulting in greater RS retention for fried potato chips [31]. The practicalities of this should, however, be considered as both longer and shorter cooking times may result in an unpalatable product. If the gelatinized starch is allowed to cool, the amylose and amylopectin chains recrystalise by a process known as retrogradation. Recrystalised or retrograded amylose is resistant to the action of small intestinal α-amylase and this now forms RS3. Raatz et al. compared baking and boiling cooking methods for three different varieties of potato and measured the RS content at three service temperatures, hot, chilled and reheated [32]. Whilst they found no significant differences between varieties, they reported more RS in baked versus boiled across all varieties, and more RS in chilled potatoes than in hot or reheated. The greatest difference reported was in the Yukon Gold variety, where the baked/chilled combination (5.4 ± SD 0.05 g/100 g) had more than double the RS of the boiled/reheated combination (2.2 ± SD 0.05 g/100 g). When potatoes are deep-fried, the resulting RS [31] may be derived from the formation of complexes between the starch and other compounds in the potato or the food matrix, such as lipids [33]. This would now be termed RS5. In one study, potato that had been boiled and cooled was compared with potato that had been boiled then deep- or shallow-fried then cooled, the amount of total RS was as follows: boiled/cooled: 1.78 ± SD 0.24% > shallow fried/cooled: 1.11 ± SD 0.05% > deep fried/cooled: 1.04 ± SD 0.13% [34]. It was hypothesized that the frying process created RS5, which inhibited RS3 formation during retrogradation, such that the fried/cooled potato had more RS5 but less RS3, resulting in less total RS than the boiled/cooled potato. It is feasible that if the potato had been cooled before the addition of oil and deep-fried later, then both RS3 and RS5 formation would be maximized, however, to our knowledge, this has not yet been tested in the potato. It should be ensured, however, that any lipid consumed remains within current dietary guidelines for content and composition, until the metabolic fate of fat ‘trapped’ in RS5 is known. To summarise, any potato product eaten will contain variable amounts of RS2, RS3 and RS5, due to variation in cooking methods, length of cooking and both cooking and storage temperatures.

#### 2.1.3. Protein and Fat

Quantitatively, potatoes are not a good source of protein, with an average content of 2–3 g/100 g (Table 1). To put this into context, the average potato intake in the UK is 85 g per capita per day [13] which we estimate would provide about 4% of the Reference Nutrient Intake (RNI) of protein for a 70 kg adult [35]. Interestingly, as recently reviewed, about 0.6 g/100 g of the protein is associated with the starch matrix in isolated potato starch, and proteomic analysis of potato starch revealed 36 different proteins [36], indicating possible targets for modifying starch biosynthesis and metabolism. The amount of fat in a potato is even less than protein. Without the addition of extra fat during preparation, the fat content in potato crop is ~0.1% of fresh weight.

### 2.2. Micronutrients

Potatoes are important sources of several micronutrients, including potassium, magnesium, vitamin C, vitamin B6, folate and thiamin. Various factors, such as variety, cooking method, and type/length of storage, affect how much of a given micronutrient is present; the effects of variety and storage will be discussed in detail in later sections. Boiling causes leaching of water-soluble vitamins and minerals. The scale of the losses are affected by duration of boiling and also surface area of the potato pieces. In one study [38], potassium losses of over 50% were observed when potatoes were cut into 1 cm cubes and boiled for 10 min, with even greater losses (70–75%) observed when the potatoes were shredded; substantial losses were also reported for iron, magnesium, manganese, phosphorus, sulphur, and zinc. These losses can be mitigated somewhat by boiling the potatoes in their skins, rather than after peeling (Table 1). Conversely, cooking methods that do not involve water preserve more of the water soluble vitamin and mineral content [39]. For example, vitamin C losses were lower when potatoes were microwaved (<33%), baked (<51%) and sautéed (<67%) than boiled (<88%) [40]. Interestingly, in the same study, addition of salt to the boiling water slightly reduced the vitamin C loss to 61–79%.

Table 1 shows the micronutrient composition of 100 g potato comparing various cooking methods with the content of raw potato. To put these figures in context, a medium-sized baked potato (200 g), for example, would contribute 24% of the UK daily reference nutrient intake (RNI) for iron for a man, 18% for magnesium, 30% for potassium, 48% for vitamin C, 44% for vitamin B6, 28% for folate and only 2% for sodium, with 14%, 10%, 30%, 48%, 52%, 28%, and 2% respectively for a woman. Furthermore, in an analysis of data from the NDNS (2008–2011), Gibson and Kurilich reported that potato consumption contributed 15% of potassium, 15% of B6, 14% of vitamin C, 10% of folate and 9% of magnesium in the UK diet [13].

### 2.3. Phytonutrients

Potatoes contain several types of phytonutrients including carotenoids, anthocyanins, and chlorogenic and caffeic acids [41] which are all antioxidants. Chu et al. analysed samples of ten different vegetables for total phenolic content, measured for antioxidant activity as gallic acid equivalents, measured by TOSC (total oxyradical scavenging capacity) assay, and anti-proliferative activity, measured in HepG_2_ cells [42]. Potatoes were reported as having approximately <40 mg gallic acid equivalents/100 g, compared to the highest measured, broccoli, which had >100 mg gallic acid equivalents/100 g. They were in the lowest group for antioxidant activity, displaying 4.86 µmol of vitamin C equiv/g of sample, compared with the highest, red pepper, which displayed 46.95 µmol of vitamin C equiv/g of sample and also displayed minimal anti-proliferative activity. They did not, however, report which variety of potato they measured, but it is likely that it was a white potato variety as they stated that the vegetables were selected based on their per capita consumption in the US. Whilst these relative amounts are quite low, a different picture emerges when actual consumption patterns are considered. Kyoung Chun et al. measured total phenolic and antioxidant content of 14 fruits and 20 vegetables and estimated per capita consumption based on data from the United States Department of Agriculture [43]. They reported that although concentrations of total phenolics and antioxidants were relatively low in potatoes, they were the highest vegetable contributor and the third highest overall, behind oranges and apples, to the US diet, due to higher amounts being consumed. Whilst dietary antioxidants demonstrate strong antioxidant and anti-proliferative action in vitro, their bioavailability is highly variable [44]. It has been suggested that, in comparison with endogenous antioxidants, they play a minor role in direct antioxidant activity and rather that their main contribution is via indirect means, such as their effects on cell signalling and gene expression [45].

### 2.4. Effects of Potato Variety on Nutrient Composition

There is a fairly narrow range of nutrients in different potato varieties and cultivars as traditional breeding strategies are not possible [9]. Thus, a narrow range of amylose content in potatoes is usually reported e.g., 20–27% amylose (*w*/*w*) of total starch depending on variety and method of determination; 23% to 43% has also been cited [17]. However, genetic modification can increase this, with reports in excess of 80% [46]. Depending on the variety of potato, the protein content varies from 1 g to 4.2 g per 100 g of potato [8,20]. The colour of potato flesh can be an indicator of nutrient content, for example, yellow fleshed potatoes contain the carotenoids lutein and zeaxanthin [47]. The antioxidant content of potatoes, particularly coloured varieties and cultivars has been well reviewed [48]. For example, potatoes with purple and red skin/flesh contain high levels of anthocyanins and have been reported to contain the highest gallic acid equivalent total phenolic content, in comparison with both yellow and white varieties [49]. The ‘golden potato’ was developed by gene modification resulting in enrichment of β-carotene (>3000 fold over the wild type), lutein (30-fold), β-β-xanthophylls (nine-fold) and α-carotene [50]. The results from an in vitro study of bioaccessibility has led to the suggestion that the golden potato could be useful in boosting the dietary intake of retinol activity equivalents and vitamin E in children and women of reproductive age in developing countries [50]. This is important since Vitamin A deficiency is the major cause of blindness in children.

### 2.5. Effects of Storage on Nutrient Composition

Potatoes are usually planted in Spring and harvested in Autumn, yet consumers require potatoes throughout the year. This means that the fresh potato purchased by the consumer may have been stored for up to a year post harvest. In order to keep potatoes at their best for such a long period of time, their environment must be tightly regulated. Typically this means, for the fresh product, keeping them at 6–10 °C, in a well-ventilated, dark, humid environment [51]. Storing potatoes at lower temperatures further inhibits sprout production, but increases reducing sugar content, which is not desirable for potatoes destined for frying as the sugars take part in the Maillard reaction, which is responsible for the browning of potatoes when fried at high temperatures. Higher amounts of reducing sugars result in an overly-brown end-product, with increased amounts of acrylamide [52]. In one study carried out in Sweden, storage of potatoes for five months, resulted in a 60% decrease in vitamin C and a 20% increase in vitamin B6 content, with no difference in the amounts of potassium, thiamin or other vitamins and minerals [53]. Cold storage (4 °C) of a number of different cultivars for seven months, resulted in reduced vitamin C content in all cultivars (mean decrease, 52%) and slightly increased total polyphenol content in two pigmented varieties [51].

## 3. Relationship between Potato Consumption and Non-Communicable Diseases

### 3.1. Obesity

There is conflicting evidence from observational studies examining potato consumption and predictors of obesity, such as increases in weight, body mass index (BMI) and waist circumference (WC). Mozaffarian et al. reported a small weight gain of 0.71 lb (95% CI: 0.53–0.89) over a four year period for every 1 serving/day increase in boiled, baked, or mashed potato, and a larger weight gain (4.11 lb; 95% CI: 3.46–4.76) for every 1 serving/day increase in French fries [54]. French fries have also been associated with weight gain in women but not in men [55]. Consumption of French fries, but not other potato types, has been associated with increased BMI [56]. Some have observed an association between total potato intake and increased WC in women [57], whereas others have found no association between total potato intake and WC [58]. Details of these studies are reported in Table 2. A systematic review of these observational studies concluded that there was no evidence for an association between potato consumption and obesity, however, there may be some evidence for an association between French fries and obesity. Overall there were few studies and those that were included were of relatively poor quality [18].

Whilst these studies, and those discussed in later sections on T2DM and CVD, attempted to control for other dietary and lifestyle factors, utilising multivariate linear regression models, it is possible that other unidentified factors are confounding their results. For example, few studies included socioeconomic status (SES) in their analysis, although other factors associated with SES, such as activity levels, fruit and vegetable intake, smoking and alcohol intake were included in most analyses. Dietary intake was measured by food frequency questionnaires (FFQs), often containing a very restricted number of food items. The use of these questionnaires whilst practical for studies involving large numbers of participants, limits the amount of information that can be obtained. When considering potato consumption they appear to have been limited to very few options, sometimes only reporting on total potato consumption [63,64,65], or grouping baked, boiled and mashed potato as a single item [66]. Where more detail is provided, it is still insufficient with regard to cooking methods. For example, boiled potatoes will differ in nutrient content, particularly fibre, depending on whether or not they are cooked and consumed with their skins on. Additionally, French fries are often cited as producing a different effect from other preparation methods, however what is classed as a French fry varies from country to country. In the US any type of potato that has been sliced into batons and fried is classed as a French fry, regardless of size of the baton, whereas in the UK and parts of Europe the size of the baton determines the name; a very thin baton would be classed as a skinny chip, a wide-cut baton as a chip and only a medium sized baton referred to as a French fry. An additional point of confusion is that in the USA and other countries, ‘chips’ would be a thin, fried potato snack sold in bags, known as ‘crisps’ in the UK. When deep-frying, the surface area-to-volume ratio affects the amount of fat absorbed, with thinner-sliced batons absorbing more oil than their thicker counterparts. Furthermore, there is no distinction between oven-baked and deep-fried French fries, two preparation methods which could vary widely in fat content.

When food intake is assessed by dietary recall methods, answers may not be very accurate, particularly if a person’s diet has changed in the intervening years. Reverse causality can also be a concern when interpreting results as it is common for people to make changes to their diet after learning they are at increased risk of a particular disease, for example reducing saturated fat intake after receiving a high cholesterol report. If these dietary changes are not made in time to affect their health, this could lead to people with apparently healthy diets being erroneously reported to be more likely to develop a particular disease.

As changes in weight, BMI and WC take place over a relatively long period of time, it would be difficult to design a well-controlled, longer-term intervention examining their effect on these markers, particularly as potatoes are not consumed alone, but within the context of a mixed diet. Instead of measuring weight gain directly, several acute studies have compared the effects of consuming different starchy carbohydrates, including potatoes, on satiety and subsequent energy intake [67,68,69,70,71,72]. A variety of methodologies have been implemented, such as matching for carbohydrate or energy content or allowing ad libitum intake; some served the carbohydrate on its own, whereas others served it within the context of a mixed meal. These studies are summarised in Table 3.

When isoenergetic portions of starchy carbohydrates are consumed, potatoes have been reported to be more satiating than pasta, rice and bread [67,70]. Furthermore, when 38 different test foods were compared, boiled potatoes were reported to have the highest satiety index of all test foods, even when compared to protein and fat-rich foods [67]. When different preparation/serving methods were compared, Leeman et al. reported both boiled and mashed potatoes to be more satiating than French fries when meals were energy matched, but not when matched for carbohydrate content [69]. This is likely due to boiled potatoes being less energy dense and, therefore, having a larger portion size than French fries when energy matched, as feelings of fullness and satiety are affected by stomach distension and capacity [73]. Indeed, a 1000 kJ portion of boiled potatoes weighs 368 g in comparison to a matched portion of French fries which weighs only 93 g [74]. Geliebter et al. compared isoenergetic (~1000 kJ) amounts of instant mashed potato, peeled baked potato, pasta, and brown rice [70]. Each test meal was accompanied by a variable amount of water designed to bring the total meal water content to 400 g, which could potentially ameliorate some of the effects of meal volume. However, despite this, they found that both potato meals reduced appetite compared to pasta and rice. This may be because water served alongside a meal has been demonstrated to have no effect on satiety in contrast to water incorporated into a meal [75].

In contrast to these studies, Diaz-Toledo et al. reported higher satiety ratings for French fries compared to an energy-matched pasta control, with no differences between baked potato or mashed potato and the pasta control for any satiety measure [72]. There were several differences between their study and the other isoenergetic studies. Their participants were given a personalised breakfast 3 h prior to the test meal and so were not fasted when they consumed the test meal. Furthermore, their test meal was a mixed meal, containing meatballs in tomato sauce, salad and dressing; the starchy test food was not consumed in isolation. The mashed potato was a pre-prepared dish, which included a comparable amount of fat to the French fries, resulting in a smaller difference in portion weight and energy density between the two. Finally the overall energy content of the test meal was 1883 kJ, almost double that of other isoenergetic studies. These differences in energy density, macronutrient content and total energy may contribute to the contradictory results from this study.

When adult participants were given ad libitum amounts of boiled pasta, rice or potatoes, along with a fixed amount of meat, and instructed to eat until they were no longer hungry, equivalent amounts of the carbohydrate element were eaten (353–372 g), however, because of the lower energy density of the potatoes, less total energy was consumed in that meal [68]. After 4 h, satiety and hunger levels had returned to baseline for those consuming the potato meal, whereas they had not for the pasta and rice meals. Plasma insulin was lower after the potato meal, with no difference between meals for glucose, despite potatoes having a reported higher glycaemic index than pasta and rice [76]. This discrepancy is likely due to the smaller amount of carbohydrate in the potato meal.

Similarly, Akilen et al. served ad libitum amounts of potato (either boiled and mashed, oven fries or French fries), boiled pasta or rice with a fixed portion of meatballs to a group of normal-weight children [71]. Lower weights of oven fries and French fries were consumed compared to pasta, however when energy intake was compared, energy from the boiled mashed potato meal was lower than all other meals. In this study, there was no difference between meals for appetite scores until they were adjusted for energy intake, which resulted in a lower mean appetite rating/kcal for the boiled mashed potato meal in the 2 h following the meal.

Evidence for a relationship between satiety scores and energy intake at a subsequent ad libitum meal is mixed. When isoenergetic portions are compared, some have reported no difference in subsequent energy intake either between different potato preparation methods or in comparison to other starchy carbohydrates, despite higher satiety scores for boiled potatoes [70,72]. Holt et al., however, reported an inverse association between satiety score and energy intake at a second meal [67] across their 38 test foods, with a tendency for a lower total energy intake across the whole day from the most satiating foods. When participants were permitted ad libitum amounts of potato, pasta or rice at a first meal, the lower energy intake and lower 4 h satiety score from the potato meal did not translate into a greater energy intake at a subsequent ad libitum sandwich meal [68].

Of the studies that measured postprandial glucose and insulin responses [67,69], neither reported any direct correlation between glycaemic or insulinaemic response and satiety score, although an indirect relationship between insulin response and satiety was suggested by Holt et al., who reported inverse associations between both insulin score and satiety score with subsequent ad libitum energy intake.

In summary, isoenergetic portions of potatoes, in particular boiled potatoes, appear to be more satiating than other starchy carbohydrates when eaten in isolation. When ad libitum consumption is permitted, less energy is consumed in mixed meals containing potato, with no compensatory increase in energy intake at a subsequent meal, despite lower satiety ratings. It should be noted that the evidence is limited as there have been few studies of this type, particularly those examining the effects of ad libitum consumption of potatoes in the context of a mixed meal. Despite this, the results from studies so far do not support a link between potato consumption and risk of overweight and obesity.

### 3.2. Type 2 Diabetes Mellitus (T2DM)

An association between total potato consumption and risk of developing T2DM has been reported [66,77,78], with the highest risk associated with consumption of French fries. Muraki et al., in an analysis of data from three prospective cohort studies [66], reported that, for every three servings/week of boiled, mashed or baked potatoes there was an increased risk of T2DM (HR, 1.04; 95% CI 1.01–1.08), with a greater risk associated with French fries (HR, 1.19; 95% CI 1.13–1.25).

It has been suggested that the high glycaemic index (GI) of potatoes may be a contributory factor, as high GI diets have been associated with an increased risk of T2DM [79,80]. GI is a measure of how much a carbohydrate-containing food raises blood glucose in relation to a control (glucose):

GI =incremental area under the 2 h glucose response curve (IAUC) for the test food ÷IAUC for glucose ×100

Foods with a GI > 70 are classed as high GI, whereas those with a GI < 55 are classed as low GI. Various factors affect the GI of the potato, such as variety, cooking method, and length of cooking. Shorter boiling times, in particular, may lead to incomplete gelatinization of the starch, with residual RS2 contributing to a lower GI. This is well demonstrated with the Carisma potato, which has been labelled low GI [81]. This is likely due to its higher onset of gelatinization temperature than other cultivars [82], as this would result in less extensive gelatinization, and therefore more resistant starch, when cooked for the same length of time as other varieties. Using GI as a predictor of a food’s effect on blood glucose is also problematic. It is calculated based on consumption of a fixed amount of CHO, usually 50 g; it does not take portion size into account. Thus, a food may have a high GI but have little effect on blood glucose because the carbohydrate density is low, resulting in a small amount of carbohydrate being consumed in a standard portion. This has led to the suggestion that glycaemic load (GL) may give a better representation of the actual effect a food has on the glycaemic response as it considers portion size along with GI:

GL =GI ×amount of available CHO in a portion (g)÷100

A GL less than 10 is considered low, with high GL categorized as a GL > 20. Typical GI and GL values, along with the amount of available CHO in a standard portion, for some common methods of preparing and serving potatoes are shown in Table 4. To put these values in context with other starchy CHO, boiled/steamed rice typically has a GI of 68–87 and a GL of 25–33 (150 g portion), depending on rice type and cooking time, whereas pasta has a GI of 51–61 and a GL of 24–29 (180 g portion) [83].

It appears from the reported GI values that GI alone cannot explain the association observed between French fries and T2DM risk, as French fries typically have a lower GI than other potato preparations. They do have a higher GL, which may partially explain the reported associations, however, other factors such as fat content and other unidentified, unhealthy lifestyle choices cannot be discounted. It should also be considered that potatoes are not usually eaten in isolation; other foods in the meal will affect the overall GI/GL of the meal. For example, serving a baked potato with cheese reduced the GI from 93 to 39 [84] and serving chicken breast, salad, and oil with mashed potato resulted in a reduction in GI from 108 to 54 compared to mashed potato served alone [85].

Furthermore, not all studies agree, with some reporting either no association [64,65,86] or an inverse association between potato consumption and development of T2DM [63,87]. Farhadnejad et al. reported a lower incidence of T2DM in those who consumed higher amounts of potatoes (55.5 g/day) compared to the lowest (7.3 g/day) consumption range [87]. A significant inverse association was observed for both total potato consumption and boiled potatoes with a trend observed for fried potatoes. These studies are summarized in Table 5.

A recent systematic review and meta-analysis [19] reported a slightly increased risk of T2DM (RR: 1.09, 95% CI 1.01, 1.18) for every 150 g/day increase in boiled, baked and mashed potatoes, with a stronger association reported for French fries (RR: 1.66, 95% CI 1.43, 1.94). The authors reported, however, that the quality of evidence was low for total potato consumption and moderate for French fries. The contradictory and limited evidence from the epidemiology does not support any recommendation to reduce total potato intake, with the possible exception of French fries, at this time. 

### 3.3 Cardiovascular Disease (CVD) and CVD Risk Factors

Several cohort studies have examined associations between potato consumption and CVD and its risk factors; a summary of these studies is presented in Table 6. Larsson et al., investigated associations between potato consumption and risk of myocardial infarction, heart failure, stroke or mortality from CVD in Swedish men and women [94]. They found no significant association between total potato intake and risk of major CVD event or mortality from CVD. Nor did they report any associations between boiled, fried, or French-fried potato consumption and any CVD outcome. In a large cohort study investigating the relationship between fruit and vegetable consumption and risk of ischemic stroke, total potato consumption was not associated with ischemic stroke risk, although individual preparation methods were not explored [95].

Studies examining the relationship between hypertension (HT) and potato intake have reported mixed results. In a Chinese cohort, total potato consumption, stir-fried and non-stir-fried potato consumption were all associated with increased risk of developing HT [96]. However, when non-potato-consumers were excluded from the analysis, higher intakes of total potato and stir-fried potato were associated with lower risk of HT. Borgi et al. also reported an association between total potato consumption and HT for those consuming ≥ 1 serving/day and an increased risk of HT for those consuming ≥ 4 servings/week of French fries. Consuming ≥ 4 servings/week of boiled, baked and mashed potatoes was associated with increased HT risk in women but not men [97]. In contrast to these two studies, Hu et al. reported no association between total potato consumption and change in blood pressure (BP) or HT risk in either the PREDIMED or SUN cohort over four years [98].

In their systematic review and dose-response meta-analysis, Schwingshackl et al. examined associations between potato consumption and risk of chronic disease [19]. They reported no association between total potato consumption and risk of coronary heart disease or stroke, even for the highest total potato intake (150 g/day). However, high consumption of French fries (150 g/day), but not other preparation methods (boiled, mashed or baked) was associated with increased risk of HT. Again, the authors noted that quality of evidence was low for boiled, mashed, and baked potatoes and moderate for French fries. They also stated that the studies’ results were confounded by only reporting total potato consumption in the majority of cases.

There have been few interventional-type studies examining the effect of potato consumption on CVD risk factors. One explanation may be that any intervention examining these measures, would have to be carried out over a longer period of time, unlike, for example, those investigating effects on postprandial glucose metabolism. Furthermore, in order to maintain energy balance, an intervention would have to remove some other component of the diet in order to incorporate potatoes. This in itself would confound the results, because any effect may be the result of what has been removed, rather than what has been added. If potatoes were added to the diet, without removing anything then overall energy intake could increase, potentially resulting in weight gain, although this was not observed in one of the studies discussed here [99].

Arterial stiffness is an independent risk factor for the development of CVD [100]. Tsang et al. explored the effects of an anthocyanin-rich potato, Purple Majesty (PM), on pulse wave velocity (PWV), a clinical measure of arterial stiffness [101]. They found that consumption of 200 g/day of PM potatoes, for 14 days, significantly reduced carotid-femoral PWV, in healthy individuals, whereas consumption of an equivalent amount of white potato had no effect. They reported no change in blood pressure, fasted glucose, insulin, triacylglycerol or HDL-, LDL-, and total cholesterol for either potato variety. They hypothesized that anthocyanins in the PM potatoes contributed to the observed results as anthocyanin intake has been associated with reduced arterial stiffness [102].

Vinson et al. also investigated the effects of the PM potato in two separate trials [99]. In an acute study, they investigated the effects of the PM potato on plasma antioxidant activity and urinary polyphenols compared to a control biscuit containing an equivalent amount of potato starch. Plasma antioxidant capacity, measured by ferric reducing antioxidant power, was non-significantly higher following the PM potato meal and urinary polyphenols were increased by 92% (*p* = 0.09 for trend) following PM consumption, compared to controls. Urinary polyphenols are a marker for polyphenol intake, with higher concentrations associated with reduced risk of HT [103]. In a second study, they investigated the effect of PM potatoes on BP in 18 individuals, 14 of whom were hypertensive, of which 13 were taking antihypertensive medication. In this crossover trial they compared the effects of four weeks consumption of PM potatoes at lunch and dinner with no potatoes for the same time period. They reported a significant (4 mmHg) reduction in diastolic blood pressure (DBP) following PM consumption, with no significant effects on systolic blood pressure (SBP), body weight, glucose, HDL- or total cholesterol and triacylglycerol. These interventional studies are reported in Table 7.

In summary, the epidemiology generally reports no associations between potato consumption and the risk of CVD, with the possible exception of HT, where some, but not all, have reported increased risk from both total potato consumption and French fries. Potatoes are a rich source of potassium, which has been associated with reduced risk of CVD [104], however, French fries are often consumed with salt which could attenuate any beneficial effect of potassium as high salt intake is associated with HT and could increase the risk of CVD [105]. In contrast, interventional studies have demonstrated some beneficial effects from an anthocyanin-rich pigmented potato variety on PWV and DBP. Whilst these results are interesting, it should be noted that these results are confined to a single pigmented potato cultivar and no effect on PWV was observed following consumption of white potatoes. Clearly, further research is required, utilizing different, commonly consumed potato cultivars, before conclusions can be drawn.

## 4. Conclusions

We have reviewed substantive literature that has investigated the health consequences of consuming potatoes. We have found that authors have not been able to sufficiently take into account the cooking method, which is a major determinant of nutrient content of the potato as eaten. In addition, no studies measured the RS content. However, evidence did suggest a positive association between obesity, risk of T2DM, and CVD and the consumption of French fries/‘chips’ in the UK. A limited number of studies investigated satiety/energy intake after the consumption of potatoes, and made specific comparisons with other starchy CHO foods. Isoenergetic portions of potatoes, particularly boiled potatoes, appeared to be more satiating when eaten in isolation. Furthermore, studies suggest that less energy is consumed if potato rather than pasta or rice is eaten as part of a mixed meal. Potatoes are a valuable source of several key nutrients and the evidence reviewed here supports their inclusion in a healthy balanced diet, in line with current dietary guidelines.

There are always limitations and caveats for determining the diet of free-living individuals but assessing the nutritional impact of potatoes consumed by their quantity alone is particularly misleading. We should, therefore, be aware of the limitations of epidemiological studies in this respect and indeed, further research, particularly randomized controlled trials, is required to understand the role of food preparation on the nutrient content of potatoes, particularly in regard to resistant starch content.

Summary points:

- The nutritional content of a medium-sized baked potato weighing 200 g can provide a significant contribution to vitamin and micronutrient needs, containing almost half of the UK daily RNI for a man for vitamin C and vitamin B6, 30% for potassium, 28% for folate, 24% for iron, and 18% for magnesium.
- The total fibre content of 4.4 g is 15% of the 30 g per day recommended for an adult. However, these figures are markedly altered by the cooking method, for example, the vitamin C content would be 50% higher in a microwaved potato, and the iron content would be reduced by over 70%.
- A major limitation when assessing the nutrient quality of the potato is that the RS content of potatoes is not included in the gold standard AOAC method for total fibre. Therefore, the total fibre content of potatoes listed in food databases underestimates actual total fibre content, and consequently the nutritional value.
- The interaction between meal components, such as starch and lipid, is a somewhat under-explored but particularly exciting and important area, as there is the potential to change the RS starch content of a meal by making simple changes to cooking methods. Considering other meal components and portion size is also important with respect to the overall GL of a meal.

## Figures and Tables

**Table 1:** Nutrient composition per 100 g potato according to cooking method.

Nutrient	Raw (Flesh and Skin)	Boiled (Flesh Only) Cooked Without Skin	Boiled (Flesh Only) Cooked in Skin	Baked (Flesh and Skin)	Microwaved (Flesh and Skin)	Oven-Baked Chips ^1^	Fried Chips ^2^	Daily RNI; (M/F)
Water (g)	79.3	77.5	77.0	74.9	72.0	64.4	38.6	-
Energy (kcal)	77	86	87	93	105	158	312	-
Protein (g)	2.1	1.7	1.9	2.5	2.4	2.8	3.3	-
Fat (g)	0.1	0.1	0.1	0.1	0.1	5.5	14.7	-
Carbohydrate (g)	17.5	20.0	20.1	21.2	24.2	25.6	41.4	-
Fibre * (g)	2.1	1.8	1.8	2.2	2.3	2.0	3.8	-
*Minerals*								
Calcium (mg)	12	8	5	15	11	12	18	700
Iron (mg)	0.81	0.31	0.31	1.08	1.24	0.57	0.81	8.7/14.8
Magnesium (mg)	23	20	22	28	27	24	35	300/270
Phosphorus (mg)	57	40	44	70	105	87	125	550
Potassium (mg)	425	328	379	535	447	478	579	3500
Sodium (mg)	6	5	4	10	8	324 **	210 **	1600
Zinc (mg)	0.30	0.27	0.30	0.36	0.36	0.35	0.50	9.5/7.0
Vitamins								
Vitamin C (mg)	19.7	7.4	13.0	9.6	15.1	8.7	4.7	40
Thiamin (mg)	0.081	0.098	0.106	0.064	0.120	0.130	0.170	1.0/0.8
Riboflavin (mg)	0.032	0.019	0.020	0.048	0.032	0.032	0.039	1.3/1.1
Niacin (mg)	1.061	1.312	1.439	1.410	1.714	2.077	3.004	17/13
Vitamin B6 (mg)	0.298	0.269	0.299	0.311	0.344	0.261	0.372	1.4/1.2
Folate (µg)	15	9	10	28	12	23	30	200
Vitamin B12 (µg)	0.00	0.00	0.00	0.00	0.00	0.00	0.00	1.5
Vitamin A (µg)	0.00	0.00	0.00	0.00	0.00	0.00	0.00	700/600
Vitamin E (mg)	0.01	0.01	0.01	0.04	0.01	0.39	1.67	-
Vitamin D (µg)	0.00	0.00	0.00	0.00	0.00	0.00	0.00	10
Vitamin K (µg)	2.0	2.2	2.2	2.0	2.0	7.4	11.2	-

Data obtained from the USDA National Nutrient Database for Standard Reference, Release April 2018 [37]; Reference Nutrient Intake (RNI) values obtained from COMA Dietary Reference Values for Food Energy and Nutrients for the United Kingdom, 1991, and the UK Scientific Advisory Committee on Nutrition (SACN); M:male; F:female; * Total dietary fibre analysed by enzymatic gravimetric methods AOAC, does not include resistant starch; ^1^ Frozen, home-prepared, also known as French Fries; ** includes sodium added during product processing; ^2^ Fast foods, fried in vegetable oil, also known as French Fries.

**Table 2:** Summary of cohort studies investigating associations between potato consumption and weight change, BMI, and waist circumference.

Reference	Study Type; Follow-Up/Duration	*n* (%F); BMI; Age (years); Criteria	Exposure; Assessment Method	Results	Potato Categories	Comments
French et al., 1994 [55]	Cross-sectional and prospective cohort (two years)	3552 (53.9%) Normal weight to obese 37.3 ± 10.7 years (F) 39.1 ± 9.8 years (M)	Participating in a workplace weight loss intervention FFQ (18 items)	A trend for an association (*p* = 0.06) between consumption of French fries/fried potatoes and higher bodyweight in women at baseline Increased consumption of French fries associated with weight gain in women	French fries and fried potatoes in a single category; no other potatoes measured	Data from the Healthy Worker Project [59] Participants were fully clothed, including shoes, for weight measurement. Time of day was not standardised. FFQ contained only 15 highest contributors to energy and fat intake; fruit and vegetable intake was not assessed.
Halkjaer et al., 2004 [58]	Cohort (six years)	2300 (49.2%) Normal weight to obese 30–60 years Of Danish origin	Habitual diet FFQ (26 items)	A weak inverse association between potato consumption and waist circumference; insignificant after adjustment for changes in obesity	Potatoes (unspecified)	Data from the MONICA1 study Intake remained largely unchanged over the time period measured
Linde et al., 2006 [56]	Cross-sectional and prospective cohort (2 years)	1801 (71.8%) Overweight and obese (BMI > 27) >18 years	Participating in weight loss intervention Block Screening Questionnaires for Fat (15 Items) and Fruit/Vegetable/Fibre (nine items) Intake	Consumption of French fries associated with higher BMI in women, but not men at baseline Increased consumption of French fries associated with increased BMI over two years for men and women No association between potatoes and BMI at baseline or over the course of the intervention	Potatoes; French fries	
Halkjaer et al., 2009 [57]	Cohort (five years)	42,696 (52.9%) BMI 20–33.5 50–64 years	Habitual diet FFQ (192 items, 21 groups)	Energy intake from potatoes was associated with five year increase in waist circumference in women	Potatoes (not including French fries)	Data from the Danish Diet, Cancer and Health Study [60] French fries were incorporated into a Snack Foods group, potatoes were in a group of their own. All analysis by group, not individual food item.
Mozaffarian et al., 2011 [54]	Three cohorts (four year intervals)	120,877 (81.3%) Non-obese at baseline 18–64 years Energy intake 900–3500 kcal/day	Habitual diet FFQ (61/131 items)	Four year weight change was positively associated with potato intake (all categories)	Total potato intake; Boiled, baked or mashed; French fries; Potato chips	Data from the Nurses’ Health Study I and II [61] and the Health Professionals Follow-up Study [62]

BMI: body mass index; FFQ: food frequency questionnaire; MONICA1: the Danish Monitoring Trends and Determinants of Cardiovascular Disease cohort.

**Table 3:** Acute studies examining the effects of potato consumption on satiety measures and energy intake.

Reference	Participants	Study Type	Test Meals	Measures	Results
Holt et al., 1996 [67]	*n* = 11–13 per group BMI 22.7 ± 0.4 22.1 ± 2.9 years	Crossover	1000 kJ portions of 38 test foods split into food groups, carbohydrate-rich group included. Along with 220 mL water. White bread as reference food.	Seven-point scale for satiety ratings	Boiled potatoes had the highest satiety score of all foods. An inverse association between satiety score and subsequent ad libitum energy intake was observed.
Erdmann et al., 2007 [68]	11 M BMI 23.5 ± 0.5 24.4 ± 0.3 years	Crossover	150 g lean pork steak, served with ad libitum amount of boiled white pasta, boiled white rice or boiled white potatoes, all in tomato sauce. Participants asked to consume foods until comfortably satiated. Ad libitum sandwich meal provided 4 h later.	VAS scores for hunger and satiety every 15 min	Comparable amounts of potato, pasta and rice consumed at first meal (353–372 g), but energy intake significantly lower for potato meal (2177 kJ) than rice (2829 kJ) and pasta (3174 kJ). Greater satiety and less hunger following pasta and rice meals during hour 4. No difference in energy consumption at second ad libitum meal (+4 h) No differences in satiety and hunger following second meal.
Leeman et al., 2008 [69] Study 1	9 M, 4 F BMI 21.8 ± 3.1 19–27 years	Crossover	Isoenergetic 1000 kJ portions of boiled potatoes, French fries or instant mashed potatoes (reconstituted with 200 or 330 g water), providing 32.5–50.3 g available CHO, all served with 250 water or milk/water mix and 150 mL tea/coffee.	Nine-point scale (painfully hungry–full to nausea)	French fries produced a lower satiety AUC than boiled potatoes over 4 h and lower satiety AUC than the small portion of mashed potato over 0–70 min.
Leeman et al., 2008 [69] Study 2	6 M, 8 F BMI 21.9 ± 2.0 20–28 years	Crossover	50 g available CHO portions of French fries and boiled potatoes, with or without 15.4 g sunflower oil (963–534 kJ), white wheat bread reference, all served with 150 water and 150 mL tea/coffee.	Nine-point scale (painfully hungry–full to nausea)	No significant differences between meals.
Geliebter et al., 2013 [70]	6 M, 6 F BMI 22.4 ± 2.0 22–30 years	Crossover	240 kcal portions (50 g CHO) of peeled baked potato, instant mashed potato, steamed brown rice and boiled pasta. White bread as control (273 kcal, 50 g CHO). Variable amount of water (180–363 g) served on the side to bring total water content of each meal to 400 g.	Scales for hunger, fullness, desire to eat and prospective consumption	Both potato meals reduced appetite compared to pasta and rice. No differences between meals on subsequent (2 h) energy intake.
Akilen et al., 2016 [71]	Study 1: 12 M, 8 F Study 2: 6 M, 6 F 11–12 years (children) normal weight	Crossover	100 g meatballs, served with ad libitum boiled mashed potatoes (from frozen, served with milk and butter), pasta (with milk, butter and cheese powder), boiled white rice (with butter and rice seasoning), oven fries or French fries. All served 4 h after a standardised breakfast.	VAS for satiety ratings	A smaller amount of oven fries and French fries was consumed than pasta. Energy intake was lower for boiled mashed potato than all other meals. No difference between meals for mean appetite scores until adjusted for energy intake. Adjusted post-meal appetite scores were lower for boiled mashed potatoes than other test meals.
Diaz-Toledo et al., 2016 [72]	16 M, 17 F BMI 22.7 ± 0.3 34.1 ± 2.4 years	Crossover	858 kJ portions of French fries (deep-fried from frozen), baked potato (pre-prepared, microwaved from frozen), mashed potato (pre-prepared, microwaved from chilled), or potato wedges (microwaved and served chilled). Pasta (boiled) as control. All served with meatballs in tomato sauce, salad and Caesar dressing (total energy from meal, 1883 kJ). All served 3 h after a standardized, personalised breakfast. Ad libitum sandwich and yoghurt meal provided 4 h after test meal.	VAS for satiety ratings (hunger, fullness, desire to eat and prospective consumption)	Higher satiety ratings (4 h AUC) for French fries, compared to pasta. Each potato meal compared to pasta meal only; no comparisons performed between potato-based meals. No difference in energy consumption at second ad libitum meal (+4 h).

AUC: area under the curve; CHO: carbohydrate; VAS: visual analogue scale

**Table 4:** Glycaemic index, glycaemic load, and available carbohydrate values for potatoes prepared according to domestic cooking methods.

Potato Variety and Cooking Method	Glycaemic Index	Glycaemic Load (150 g Portion)	Available CHO (g per 150 g Portion)
Charlotte (waxy), boiled 15 min	66	15	23
Nicola (waxy), boiled 15 min	58–59	9	16
Carisma (waxy), boiled 8–9 min	53	8	16
Desiree, boiled 35 min	101	17	17
Pontiac, boiled 35 min	88	16	18
Russet Burbank, unpeeled, microwaved for 18 min	77 ± 9	19	25
White with skin, baked	69	19	27
Instant mashed potato	79–97	16–19	20
Desiree, mashed	102	26	26
Pontiac, mashed	91	18	20
French fries, baked 15 min	64	21	32
Irish potato, peeled, fried in oil	70	21	30

Data taken from “International Tables of Glycemic Index and Glycemic Load Values: 2008” [83], except Carisma cultivar [81].

**Table 5:** Summary of cohort studies investigating associations between potato consumption and T2DM risk.

Reference	Study Type; Follow-Up/Duration	*n* (%F); BMI (kg/m^2^); Age (years); Criteria	Exposure; Assessment Method	Results	Potato Categories	Comments
Salmerón et al., 1997 [88]	Cohort (six years)	42,759 (0%); normal weight to obese; 40–75 years Energy intake 400-4200 kcal/day	Habitual diet FFQ (131 items)	Consumption of French fries, but not total potato intake, was associated with increased risk of T2DM	Cooked potato; French fries	Data from the Health Professionals Follow-up Study [62]
Salmeron et al., 1997 [78]	Cohort (six years)	65,173 (100%); normal weight to obese; 40–65 years Energy intake 600–3502 kcal/day	Habitual diet FFQ (134 items)	Intake of both total potatoes and French fries was associated with increased risk of T2DM	Cooked potato; French fries	Data from the Nurses’ Health Study [61]
Hodge et al., 2004 [64]	Cohort (four years)	31,641 (59%); normal weight to obese; 27–75 years	Habitual diet FFQ (121 items)	Total potato intake was not associated with risk of T2DM Total carbohydrate intake was inversely associated with T2DM incidence High dietary GI was associated with increased risk of T2DM	Total potato intake	Data from The Melbourne Collaborative Cohort Study [89]
Liu et al., 2004 [65]	Cohort (8 to 9 years)	38,018 (100%); normal weight to obese; ≥45 years	Habitual diet FFQ (131 items)	Total potato intake was not associated with risk of T2DM	Total potato intake	Data from the Women’s Health Study [90]
Halton et al., 2006 [77]	Cohort (20 years)	84,555 (100%); normal weight to obese; 30–55 years at baseline; Energy intake 500–3500 kcal/day	Habitual diet FFQ (61 items at baseline, rising to 131 items), repeated assessment	Baked or mashed potato intake was associated with risk of T2DM in obese women only Intake of French fries was associated with increased risk of T2DM for all women	French fries; Baked or mashed	Data from the Nurses’ Health Study [61] Potato and French fries consumption patterns did not change over time
Villegas et al., 2007 [63]	Cohort (4.6 years)	64,227 (100%); normal weight to obese; 40–70 years	Habitual diet FFQ (77 food items/groups)	Potato consumption associated with lower risk of T2DM	Total potato intake	Data from the Shanghai Women’s Health Study [91] Study population did not consume much potato (median intake 8.1 g/day); their main CHO was rice
Von Ruesten et al., 2013 [86]	Cohort (eight years)	23,531 (61%); normal weight to obese; 35–65 years Energy intake 800–6000 kcal/day	Habitual diet FFQ (148 food items)	No associations between potato or fried potato consumption and T2DM risk	Potatoes (potatoes, mashed, potato dumpling, potato salad); Fried potatoes (French fries, croquettes, fried potatoes, potato pancake)	Data from the EPIC Potsdam Study [92]
Muraki et al., 2016 [66]	Three cohorts (four years)	199,181 (80%) Normal weight to obese; 40–75 years;	Habitual diet FFQ (61/131 items)	Consumption of potatoes, especially French fries was associated with increased risk of T2DM	Total potato intake; Boiled, baked or mashed; French fries	Data from the Nurses’ Health Study I and II [61] and the Health Professionals Follow-up Study [62]
Farhadnejad et al., 2018 [87]	Cohort (six years)	1981 (53.8%) normal weight to obese 38.9 ± 13.4 years		Total potato and boiled potato consumption both associated with lower risk of T2DM	Total potato intake; Boiled potatoes;Fried potatoes	Data from the Tehran Lipid and Glucose Study [93] Median intake 22.4 g/day

**Table 6:** Summary of cohort studies investigating associations between potato consumption and cardiovascular disease.

Reference	Study Type; Follow-Up/Duration	*n* (%F); BMI; Age (years); Criteria	Exposure; Assessment Method	Results	Cooking Methods	Comments
Joshipura et al., 1999 [95]	Two cohorts (NHS: eight years; HPFS: 14 years)	114,276 (66%); mean BMI 24.3–25.45 kg/m^2^; M: 40–75 years F: 34–59 years; CVD-, cancer- and T2DM-free at baseline	Habitual diet FFQ (61/131 items)	No association between potato consumption and ischemic stroke risk	Not Specified	Data from the Nurses’ Health Study I and II [61] and the Health Professionals Follow-up Study [62]
Larsson et al., 2016 [94]	Two cohorts (13 years)	69,313 (47.3%); M: 45–79 years F: 49–83 years; CVD-, cancer- and T2DM-free at baseline	Habitual diet FFQ (96 items)	Neither total potato consumption nor any individual cooking method was associated with risk of major CVD events (myocardial infarction, heart failure, stroke) or mortality from CVD	Total potatoes; Boiled potatoes; Fried potatoes; French fries	Data from the Cohort of Swedish Men and the Swedish Mammography Cohort Median total potato consumption 4.5–5.5 times/week, mainly from boiled potatoes (3.5 times/week)
Borgi et al., 2016 [97]	Three cohorts (max 24–34 years) Health questionnaires every two years	187,453 (80.4%) Non-hypertensive at baseline BMI 20.9–31.8 kg/m^2^ F: 25–55 years M: 40–75 years	Habitual diet FFQ (61/131 items)	≥1 serving/day of potato (all types) associated with increased risk of hypertension, compared to <1 serving/month ≥4 servings/week of boiled, baked or mashed potatoes associated with increased risk of hypertension in women but not men, compared to <1 serving/month ≥4 servings/week French fries associated with increased risk of hypertension, compared to <1 serving/month	Total potato intake; Boiled, baked or mashed; French fries; Potato chips	Data from the Nurses’ Health Study I and II [61] and the Health Professionals Follow-up Study [62]
Hu et al., 2017 [98]	2 cohorts 4 and 6–7 years follow-ups)	PREDIMED: 6940 55–80 years CVD free, but at high risk (T2DM or ≥3 of: smoking, hypertension, high LDL, low HDL, overweight, family history of CVD) SUN project: 13,837 M: 42.7 ± 13.3 years F: 35.1 ± 10.7 years	PREDIMED: Mediterranean diet FFQ (137 items) SUN: Habitual diet FFQ (136 items)	Total potato intake not associated with change in BP or incidence of hypertension over 4 years	PREDIMED: Potato chips (crisps), homemade fries, cooked or boiled potatoes SUN: Fried potatoes, cooked or roasted potatoes	Data from the PREDIMED [106] and SUN [107] cohorts
Huang et al., 2018 [96]	Cohort Mean 11.3 years	11,763 (54.6%) 20–93 years No hypertension, infarction or diabetes at baseline	Habitual diet, three day dietary recall	Sweet potato associated with HT in urban residents Potatoes (*p* = 0.1225), stir-fried potatoes (*p* = 0.2168) and non-stir-fried potatoes (*p* = 0.0456) all associated with HT When non-potato consumers were excluded, higher consumption of total potatoes and stir-fried potatoes associated with lower risk of HT	Total potatoes; Sweet potatoes; Stir-fried potatoes; Non stir-fried potatoes	Data from the China Health and Nutrition Survey [108] Urban residents more likely to consume sweet potato in snack form (fried chips, sugar-cured fries) Rice was the main starchy CHO in the diet, potato consumption was much lower than Western countries; potatoes were more often consumed as a side dish

HDL: high density lipoprotein cholesterol; HPFS: Health Professionals’ Follow-up Study; HT: hypertension; LDL: low density lipoprotein cholesterol; NHS: Nurses’ Health Study; PREDIMED: PREvención con DIeta MEDiterránea; SUN: Seguimiento University of Navarra.

**Table 7:** Summary of intervention studies investigating effects of potato consumption on cardiovascular disease risk factors.

Reference	Study Type; Follow-up/Duration	*n* (F); BMI; age (years); Criteria	Exposure; Assessment Method	Results	Cooking Methods	Potato Type	Comments
Vinson et al., 2012 [99]	1. RCT: Single meal 2. Supplementation: Crossover (four weeks)	8 (1); 5 normal weight, 2 overweight, 1 obese; 23 ± 9 years; Healthy 18 (11); 5 normal weight, 6 overweight, 7 obese; 54 ± 10 years; 14/18 hypertensive; 13/18 taking BP lowering medication	6–8 small potatoes (~138 g), microwaved; Biscuit containing equivalent amount of potato starch served as control; Plasma samples (0, 0.5, 1, 2, 4, and 8 h) 24 h urine collection (urine polyphenols), pre- and post-study 6–8 small potatoes, microwaved, twice a day (lunch and dinner); No potatoes consumed on alternate arm; (BP, body weight, glucose, HDL, TAG, TC) pre/post trial	Non-significantly lower plasma antioxidant capacity following control meal, compared to potatoes (*p* = 0.11) Urine polyphenols increased by 92% following potato consumption and decreased by 3.5% following control biscuit (*p* = 0.09 for trend) 4 mmHg reduction in DBP following potato supplementation (*p* < 0.01); No effect on plasma glucose, HbA1c or lipids No effect on SBP or body weight	Microwaved, consumed with skins	Purple majesty (pigmented potato)	Participants followed a low polyphenol diet for three days prior to the acute study
Tsang et al., 2018 [101]	RCT (14 days, with seven days washout between treatments)	14 (8F); BMI: 19.4–31.2 kg/m^2^ (11 normal weight, 2 overweight, 1 obese) 20–55 years; Healthy; Normotensive	200 g/day of PM potato versus white potato control; PWV, BP, bodyweight Plasma samples (TAG, HDL, LDL, TC, CRP, insulin, glucose)	Consumption of PM potatoes, but not white potatoes, significantly reduced PWV; No changes in any other measure for either PM or white potato	Boiled with skin	Purple Majesty (PM)	PM potatoes contain significantly higher amounts of anthocyanins than white potatoes (control); Participants were forbidden certain high-polyphenol foods and drinks and advised to limit fruit, vegetable and potato intake during the study

CRP: C-reactive protein; TAG: triacylglycerol; TC: total cholesterol.
